# Immunoglobulin therapy of abdominal sepsis in emergency surgery

**DOI:** 10.1186/cc11691

**Published:** 2012-11-14

**Authors:** O Butyrsky, V Starosek

**Affiliations:** 1 Crimean Medical University, Simferopol, Ukraine

## Background

The literature data demonstrate that the initial stage of abdominal sepsis (AS) is characterized with typical immune shifts as white blood cell (WBC) activation, decrease of T-lymphocyte and B-lymphocyte function followed by dysimmunoglobulinemia. As AS progresses immunity deficiency acquires severe combined character. Impact of immunity deficiency on a course is not described.

## Methods

We investigated 33 patients (male:female = 25:8) with abdominal sepsis (total peritonitis following appendicitis, perforated duodenal ulcer, pancreonecrosis). Anti-endotoxin (AE) antibodies (anti-LPS-IgA, anti-LPS-IgM, anti-LPS-IgG) were determined by original modification of hard-phase immunoenzyme analysis. *Escherichia coli K30 *LPS was used as antigen for AE antibody detection. All data were compared with healthy donors (99 patients). As a method of immunity correction we selected Sandoglobulin (Novartis, Switzerland), introduced as 3 ml once intravenously.

## Results

According to results of immunity parameter investigation on the day of admission we shared all patients into two groups: with high initial immunity (*n *= 6, no evidence from normal parameters) and low initial immunity (*n *= 27, evident decrease of AE immunity parameters). All patients needed comprehensive medication support. The patients with high immunity did not need any immunotherapy. But the patients with low initial immunity required immune therapy to avoid severe course of peritonitis and unfavorable outcomes. Single introduction of Sandoglobulin on the fifth day after surgery was accompanied with increase of immunity indices (Table 1). Simultaneously with an increase of anti-LPS-immunoglobulin titer one noticed sharp positive clinical changes (improvement of patients' self-feeling, body temperature decrease, decrease/normalization of WBC number, neutrophil shift). We have the following data about routine laboratory parameters in abdominal sepsis patients with Sandoglobulin introduction: WBC, ×10^9^/l, 16.5 ± 0.9 before introducing and 9.8 ± 1.2 in 2 days after introducing; stab neutrophils, %, 18.3 ± 1.6 and 10.8 ± 1.4 appropriately; juvenile neutrophils, %, 4.1 ± 0.8 and 0.3 ± 0.1 appropriately (all changes are evidently proved, *P *< 0.001). Successful surgical treatment and immunotherapy of abdominal sepsis are accompanied by activation of AE immunity, all-class anti-LPS-immunoglobulin concentration growth that blocks mechanisms of further inflammation progress and result in rapid patients' recovery.

**Table 1:** Dynamics of AE antibodies in abdominal sepsis patients with Sandoglobulin introduction

	Patients with low immunity level (*n *= 27)	
	
	Before introduction (optic units)	2 days after introduction (optic units)
Anti-LPS-IgA	0.154 ± 0.015	0.342 ± 0.02*
Anti-LPS-IgM	0.213 ± 0.01	0.284 ± 0.02*
Anti-LPS-IgG	0.083 ± 0.007	0.186 ± 0.04*

*P < 0.001.

## Conclusion

Peritonitis patients with initial low immunity need passive nonspecific immunotherapy that stimulates organism protective functions and promotes rapid recovery.

